# Supplementary material for: The Prevalence and Determinants of Vitamin D Status in Community-Dwelling Older Adults: Results from the English Longitudinal Study of Ageing (ELSA)
Source: Nutrients. 2019 Jun 1;11(6):1253. doi: 10.3390/nu11061253 (PMC6627050; doi:10.3390/nu11061253)
Supplement: Supplementary file 1 [file nutrients-11-01253-s001.pdf]

Table 1. Participant characteristics stratified by vitamin D deficiency (IOM and ES).

	**Overall (*n* = 6004)**	**<30 nmol/L (*n* = 1423)**	**<50 nmol/L (*n* = 3317)**
	N (%) ± SD	N (%) ± SD	N (%) ± SD
	**Socio-demographic factors**		
**Age, years**	66 4 ± 8 8	66·4 ± 9 5	66·3 ± 9 1
50–59	1476 (24·6)	399 (28·0) ***	865 (26·1) **
60–69	2463 (41·0)	528 (37·1) ***	1305 (39·3) **
70–79	1553 (25·9)	335 (23·5) *	830 (25·0)
80+	512 (8·5)	161 (11·3) ***	317 (9·6) ***
**Female**	3291 (54·8)	832 (58·5) **	1855 (55·9)
**Education** ≥ O-level	4392 (73·2%)	979 (68·8) ***	2382 (71·8) **
No qualification	1340 (22·4)	383 (26·9) ***	554 (20·6) **
**Marital Status**			
[a] Married	3997 (66·6)	830 (58·3) ***	1925 (71·6) ***
Single	320 (5·3)	96 (6·8) **	121 (4·5) **
Widow	763 (12·7)	240 (16·9) ***	283 (10·5) ***
	**Health and Lifestyle factors**		
**BMI kg/m^2**	28·0 ± 4·9	28·9 ± 5·6 ***	27·2 ± 4·4 ***
Underweight	50 (0·9)	17 (1·2)	26 (0·9)
Normal	1578 (27·1)	311 (21·9) ***	815 (31·2) ***
Overweight	2461 (42·3)	501 (35·2) ***	1180(45·1) ***
Obese	1731 (29·7)	521 (36·6) ***	595 (22·7) ***
**Physical Activity**			
Vigorous (>1/wk)	1359 (22·6)	194 (13·6) ***	773 (28·8) ***
Moderate (>1/wk)	3933 (65·6)	768 (53·9) ***	1917 (71·3) ***
Mild (>1/wk)	4975 (82·9)	1114 (78·3)***	2285 (85·0) ***
**Current smoker**	695 (11·6)	267 (18·8) ***	213 (7·9) ***
**Alcohol,** 5–6day/wk	356 (5·9)	62 (4·4) **	188 (6·9) **
No alcohol	646 (10·8)	243(17·1) ***	423(12·8) ***
**SR Health**			
Excellent	772 (12·9)	122 (8·6) ***	436 (16·2) ***
Very good	1908 (31·8)	368 (25·9) ***	898 (33·4) **
Good	1901 (31·8)	442 (31·1)	853 (31·7)
Fair	1062 (17·7)	332 (23·3) ***	387 (14·4) ***
Poor	360 (6·0)	159 (11·2) ***	114 (4·2) ***
[b] **Limiting illness**	1887 (31·4)	554 (38·9) ***	735 (27·4) ***
**Polypharmacy**	1546 (25·7)	436 (30·6) ***	932 (28·1) ***
	**Predictors of Vitamin D Status**		
**25(OH)D,** nmol/L	48·7 ± 23·4	21·3 ± 5·1	31·7 ± 10·6
Winter blood	3564 (59·4)	1009 (70·9) ***	1354 (50·4) ***
[c] Sun travel	3457 (57·6)	663 (46·6) ***	1676 (62·4) ***
VitD supp user	262 (4.4)	27 (10.3) ***	72(27.5) ***

Notes: SD, standard deviation; kg/m^2, kilograms per metre squared; SR, self-reported; winter, October to March. [a] includes those first time, remarried and legally recognised civil partnership. [b] sun holiday within the last 12 months. [c] If longstanding illness is limiting. Independent Student t-test and χ^2 comparison between groups defined as deficiency <30 nmol/L versus ≥30 nmol/L and <50 nmol/L versus ≥50 nmol/L. *P* value denoting significant levels between groups $p < 0.05$*, $p < 0.01$**, $p < 0.001$***.

Table S2. Serum 25(OH)D status by region in England, findings from the ELSA Study (n = 6004). Prevalence (%) vitamin D deficiency (IOM, <30 nmol/L) and vitamin D deficiency (ES, <50 nmol/L), stratified by season [a] and region.

**Region**	**Overall (n = 6004)**	**°N Latitude**	**Mean 25OHD**	**SD**	**Vitamin D deficiency <30 nmol/L**			**Vitamin D deficiency <50 nmol/L**		
					**Overall N = 1423 (23·7%)**	**Winter N = 1009 (28·3%)**	**Summer N = 414 (16·9%)**	**Overall N = 3316 (55.2%)**	**Winter N = 2210 (62.0%)**	**Summer N = 1106 (45.2%)**
North East	375	54·9	46·4	23·3	28·8	36·4	20·6	61·6	70·3	52·2
Yorkshire & H	656	53·7	47·9	22·0	25·9	28·9	14·8	56·3	60·3	48·7
North West	661	53·6	47·5	23·9	25·9	29·4	20·3	60·2	66·4	51·3
East Midlands	658	52·8	48·1	23·0	24·9	30·5	17·6	54·6	62·0	44·7
West Midlands	655	52·5	45·4	24·9	29·8	24·5	21·3	61·2	68·1	48·9
East England	723	52·2	50·6	23·7	29·6	31·3	16·4	52·0	56·5	45·6
London	483	51·5	45·6	23·7	29·6	31·3	26·4	62·1	65·9	54·6
South East	1047	51·1	52·3	23·5	17·9	21·2	13·8	48·9	56·0	39·7
South West	746	50·4	50·6	22·1	19·6	26·6	10·3	49·6	60·7	34·9

Notes: [a] Season defined by vitamin D calendar; summer April-October.

Table S3. Weighted logistic regression for predictors of vitamin D deficiency (<50 nmol/L) in ELSA study participants (n = 6004).

	Unadjusted Model 2		Adjusted Model 2	
**Demographic variables**	**OR**	**(95% CI)**	**OR**	**(95% CI)**
**Female**	1·06	(0·93, 1·20)	1·08	(0·78, 1·24)
**Age** 50–59	1		1	
60–69	0·76***	(0·65, 0·88)	0·94	(0·78, 1·12)
70–79	0·82*	(0·69, 0·96)	1·03	(0·83, 1·29)
80+	1·28*	(1·00, 1·64)	1·67***	(1·23, 2·28)
**Non-white ethnicity**	4·38***	(2·57, 7·44)	4·67***	(2·57, 8·51)
**Marital Status**—Married	0·72***	(0·63, 0·81)	0·89	(0·78, 1·04)
Widow	1·46***	(1·22, 1·74)	1·27*	(1·01, 1·60)
Single	1·39**	(1·08, 1·81)	1·29	(0·94, 1·77)
**Retired**	0·78***	(0·69, 0·89)	0·73***	(0·63, 0·86)
**Education**—O-level min.	1·22**	(1·06, 1·41)	0·93	(0·79, 1·09)
**Region-** North	1		1	
Midlands	0·84*	(0·72, 0·98)	0·83*	(0·69, 0·97)
South	0·81**	(0·69, 0·94)	0·79**	(0·66, 0·93)
[a] **Latitude** (°N)	1·09***	(1·04, 1·15)	1·09***	(1·04, 1·15)
**Modifiable Health and Lifestyle factors**				
**BMI**- normal	0·68***	(0·59, 0·79)	0·81**	(0·69, 0·96)
obese	1·78***	(1·54, 2·05)	1·53***	(1·30, 1·79)
**Alcohol intake** 5–6 week	0·72**	(0·56, 0·94)	0·98	(0·75, 1·28)
3–4week	0·65***	(0·55, 0·78)	0·77**	(0·64, 0·95)
**Current smoker**	1·97***	(1·59, 2·42)	1·8***	(1·45, 2·25)
**SR Health**—Excellent	1		1	
Very good	1·36**	(1·09, 1·68)	1·21	(0·97, 1·52)
Good	1·59***	(1·29, 1·96)	1·29*	(1·02, 1·62)
Fair	2·24***	(1·78, 2·82)	1·59***	(1·23, 2·05)
Poor	2·48***	(1·79, 3·43)	1·55*	(1·09, 2·23)
**VitD supplement use**	0·32***	(0·23, 0·43)	0·24***	(0·18, 0·33)
**Sun travel**	0·66***	(0·58, 0·75)	0·86*	(0·75, 0·99)
**Season** (summer)	0·51***	(0·45, 0·58)	0·47***	(0·41, 0·54)
**PA**—Vigorous (>1/wk)	0·54***	(0·46, 0·63)	0·69***	(0·59, 0·83)
Moderate (>1/wk)	0·62***	(0·55, 0·71)	0·86	(0·74, 1·01)

Notes: OR, odds ratios; CI, confidence interval; SR, self-reported; PA, physical activity. For the adjusted model, variables included socio-demographics (age in years, gender, and region of residence), health-related behaviours (current smoker, vitamin D supplement use), physical factors (vigorous PA >1/week, BMI), and season (winter/summer blood sampling). All variables were included and e assessed using the same categorisation/groupings, for visual presentation only categories that reached statistical significance are shown. [a] Replaced region in the adjusted model. P value denoting significant levels between groups $p < 0·05$*, $p < 0·01$**, $p < 0·001$***.

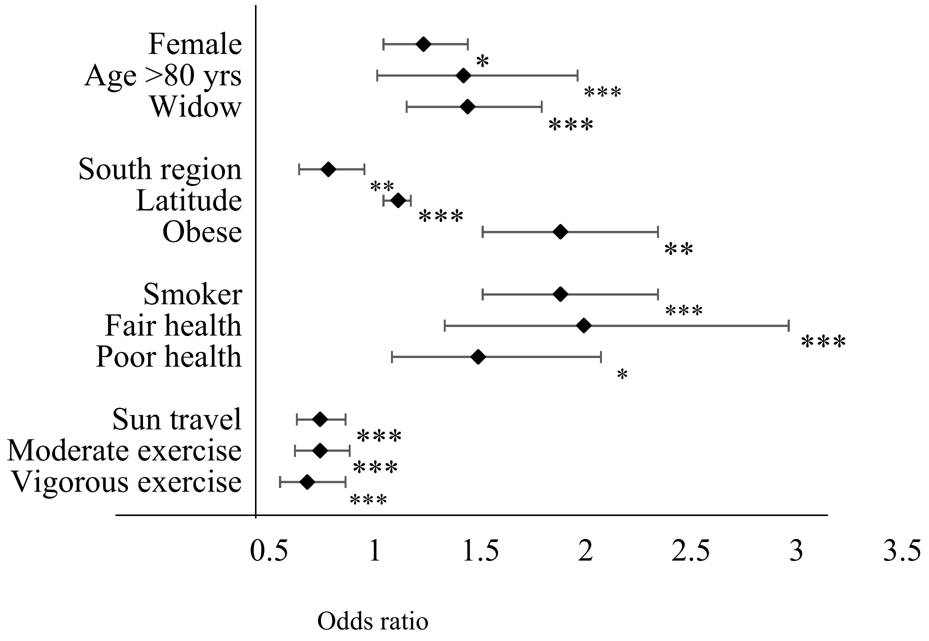

Figure 1. Weighted logistic regression for selected predictors of vitamin D deficiency (IOM <30 nmol/L) in ELSA study participants. *P*-value denoting significant levels between groups $p < 0.05$ *, $p < 0.01$ **, $p < 0.001$ ***.
